# Differences in image density adjustment parameters on the image matching accuracy of a floor‐mounted kV X‐ray image‐guided radiation therapy system

**DOI:** 10.1002/acm2.13505

**Published:** 2021-12-20

**Authors:** Kanako Sakuragawa, Motoharu Sasaki, Takeshi Kamomae, Michihiro Yokoishi, Ryosuke Kasai, Akimi Kajino, Hitoshi Ikushima

**Affiliations:** ^1^ Department of Radiological Technology Tokushima University Hospital Tokushima 770‐8503 Japan; ^2^ Department of Therapeutic Radiology Institute of Biomedical Sciences Tokushima University Graduate School Tokushima 770‐8503 Japan; ^3^ Department of Radiology Nagoya University Graduate School of Medicine Nagoya Aichi 466‐8550 Japan; ^4^ Graduate School of Health Sciences Tokushima University Tokushima 770‐8503 Japan

**Keywords:** brain metastasis, digitally reconstructed radiograph, ExacTrac, stereotactic radiosurgery

## Abstract

This study aimed to investigate the effect of two different image density adjustment parameters on the results of image matching at six degrees of freedom using radiographic images generated by the ExacTrac X‐ray system in brain stereotactic radiosurgery (SRS). This study comprised 32 patients who underwent brain SRS at our hospital from January 2020 to December 2020. In this study, (1) the default parameter (an image density parameter between “tissue” and “bone”) was an image density parameter for digitally reconstructed radiograph (DRR) generation used at many facilities, and (2) the bone parameter was the steepest contrast parameter used at our hospital. Of the 32 patients, 24 (75%) had a couch angle of 0.5 mm or more in the translational direction or 0.5° or more in the rotational direction, and 10 (31%) had a couch angle of 1.0 mm or more in the translational direction or 1.0° or more in the rotational direction. Among the 131 cases of all couch angles, 46 (35%) cases had a translational direction of 0.5 mm or more or a rotational direction of 0.5° or more, and 15 (11%) had a translational direction of 1.0 mm or more or a rotational direction of 1.0° or more. The results of this study indicate the usefulness of using appropriate DRR parameters for each case, rather than using the default settings. The use of appropriate DRR parameters can lead to accurate position matching results, leading to fewer image‐guided radiation therapy shots and a lower imaging dose.

## 1 INTRODUCTION

Stereotactic radiosurgery (SRS) is a well‐established technique for treating benign and malignant brain lesions, and several studies have reported that SRS yields satisfactory clinical outcomes.^1^, ^2^, ^3^ The stereotaxic frame has been proposed as an invasive head ring for precise fixation of the target lesion,^4^ and it has also been adopted in SRS using a linear accelerator (LINAC).^5^ Although stereotaxic frames ensure high fixation accuracy, they have the disadvantage of causing pain and discomfort to the patient. Recently, a noninvasive stereotaxic system has been developed and adopted.^6^ In addition, image‐guided radiation therapy (IGRT) has become widespread, which has further improved patient setup accuracy.

IGRT plays an essential role in brain SRS because it supports identification of accurate targets and avoidance of organs at risk.^7^ The methods of IGRT for brain SRS identified in the web survey are reported to be used in the following order: kV or MV cone beam computed tomography (CBCT), two‐dimensional (2D) imaging, and ExacTrac X‐ray system (ETX) (Brainlab AG, Munich, Germany).

Different from other IGRT systems, the ETX is independent of the LINAC and can be located and corrected using the noncoplanar method.^8^ Reports on the accuracy of the ETX indicate that the overall positional accuracy is within 0.5 mm.^9^, ^10^, ^11^ In addition, many previous studies have reported the patient setup accuracy of brain localization using the ETX,^12^, ^13^, ^14^, ^15^, ^16^ showing that highly accurate positioning is possible. Many recent reports on patient setup accuracy using the ETX are based on six‐degree‐of‐freedom (6DOF) fusion algorithms.^12^, ^13^, ^14^, ^16^ Different from 2D imaging and CBCT, 6DOF image matching with X‐ray images generated by the ETX does not require manual image matching. The ETX radiographic 6DOF image matching can only be performed automatically. Therefore, the results of image matching in 6DOF using X‐ray images of the ETX may vary depending on the digitally reconstructed radiograph (DRR) generation parameters and image quality due to the slice thickness and imaging conditions during computed tomography (CT) for treatment planning. Yan et al.^17^ have reported that differences in image quality due to CT slice thickness and imaging conditions affect the accuracy of image matching using the Rando phantom. It has been reported that the accuracy between CT slice thicknesses of 2 and 5 mm varies little in the left–right (L‐R) and anterior–posterior (A‐P) directions, with the highest localization accuracy in the superior–inferior (S‐I) direction at a slice thickness of 2 mm.^17^ The DRRs used in the image fusion are moved and rotated horizontally and vertically with respect to the X‐ray images captured by the ETX. In order to perform image fusion, a number of DRR patterns are generated from the treatment plan CT images captured in the ETX workstation beforehand by using the full arithmetic functions of the graphics board and performing high‐speed rendering processing. Therefore, it is known that if the look‐up table or gamma setting is changed the subsequent image fusion will be altered, and thus the correction shift is calculated.^18^ However, the ETX user manual states that in most cases it is not necessary to change the default values. Furthermore, to the best of our knowledge, there is no previous study reporting on the effect of different image density adjustment parameters, one of the DRR generation parameters, on the results of image matching in 6DOF using the ETX.

This study aimed to investigate the effect of two different image density adjustment parameters on the results of image matching at 6DOF using radiographic images generated by the ETX in brain SRS. To reduce the frequency of cerebral necrosis, many facilities that perform brain SRS use a setup margin of 1–2 mm, and the accuracy of position matching is critical because the margin of 1–2 mm is more severe than that of other regions and because of the lengthy treatment duration. Therefore, by reducing the uncertainty of position matching in brain SRS, it will be possible to reduce the number of imaging matches. Furthermore, if we can reduce the number of acquisition matches, the imaging dose can be naturally reduced. Therefore, we believe that this study provides helpful information for ETX users.

## 2 MATERIALS AND METHODS

### 2.1 Patients and clinical plan

Eligible cases included 32 patients who underwent stereotactic brain surgery at our hospital from January 2020 to December 2020. Written consent was obtained for the use of all imaging data for research purposes. Treatment planning was performed using Eclipse (Varian Medical Systems, Palo Alto, CA, USA) version 11.0.31, with a CT slice thickness of 1.25 mm. The average (minimum to maximum) couch angles for each of the four arcs were 32° (15°–45°), 68° (55°–80°), 295° (285°–315°), and 334° (330°–345°). The clinical target volume margin to the planning target volume (PTV) is set at 2 mm at our hospital. The prescribed dose at our hospital is 18–20 Gy per fraction, defined as the dose containing 95% of the PTV volume (D95%). Patients undergoing brain SRS were fitted with RT‐1889 (Q‐Fix, Avondale, PA, USA), a U‐shaped thermoplastic mask with a thickness of 3.2 mm.

### 2.2 Patient setup

The yaw direction of the patient setup was determined using markers attached to the thermoplastic mask during treatment planning and a laser in the radiotherapy room. Patient setups were adjusted in the L‐R, S‐I, and A‐P directions for fine positioning according to a submillimeter scale using “plastic infrared body markers” that could be identified by the infrared (IR) camera attached to the ETX.

### 2.3 Patient location matching method

After patient setup, X‐ray images were taken with the ETX at a 0° couch. Subsequently, the couch was corrected by 6DOF based on the image matching results. Next, to evaluate and confirm the accuracy of the couch correction by 6DOF (target registration error (TRE)), X‐ray images were retaken using the ETX at a 0° couch. The allowable TRE values were within 0.5 mm for the translational directions of the L‐R, S‐I, and A‐P directions and within 0.5° for the three rotational directions of yaw, roll, and pitch.

Subsequently, we moved to the actual irradiation couch angle and performed image matching for each irradiation couch angle. The couch was corrected by 6DOF until the results of image matching by X‐ray images using the ETX were obtained within 0.5 mm in the translational direction and 0.5° in the rotational direction at any irradiation couch angle. For the image density parameter, one of the DRR generation parameters used for image matching, “bone,” was selected as the steepest contrast.

### 2.4 ETX system and image matching method/workflow

**FIGURE 1:**
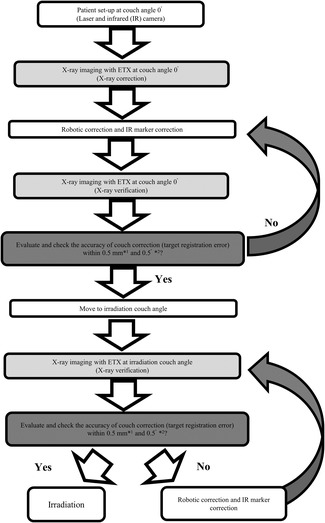
Flowchart of position matching and irradiation in our facility. *1: the translational directions (left–right, superior–inferior, and anterior–posterior) were within 0.5 mm. *2: the axial rotation directions (yaw, roll, and pitch) were set within 0.5° Abbreviation: ETX, ExacTrac X‐ray system.

The patient was set up using a laser and an IR camera at a 0° couch, and X‐ray images were obtained using ETX (X‐ray correction). Then, the couch was corrected by 6DOF based on the image matching results. In order to evaluate and confirm the accuracy of the couch correction by 6DOF (TRE), X‐ray was again performed following the same conditions (X‐ray verification). A translational direction of 0.5 mm or less and 0.5° or less in the three rotational axis directions were allowed for TRE. In case these allowable values were exceeded, a couch correction by 6DOF and position correction by IR marker were performed until the allowable values were reached. After that, the couch was rotated to the actual irradiation couch angle, and image matching was performed for each irradiation couch angle. As in the case of the actual irradiation couch angle of 0°, image matching for each irradiation couch angle was also performed within 0.5 mm for the three axes in the translational direction and within 0.5° for the three axes in the rotational direction. If the allowable value was exceeded, the actual irradiation was not performed until the allowable value is reached by performing couch correction using 6DOF and position correction using IR markers again. A flowchart of position matching and irradiation at our hospital is shown in Figure 1.

### 2.5 Difference in position matching by image density parameters

**FIGURE 2:**
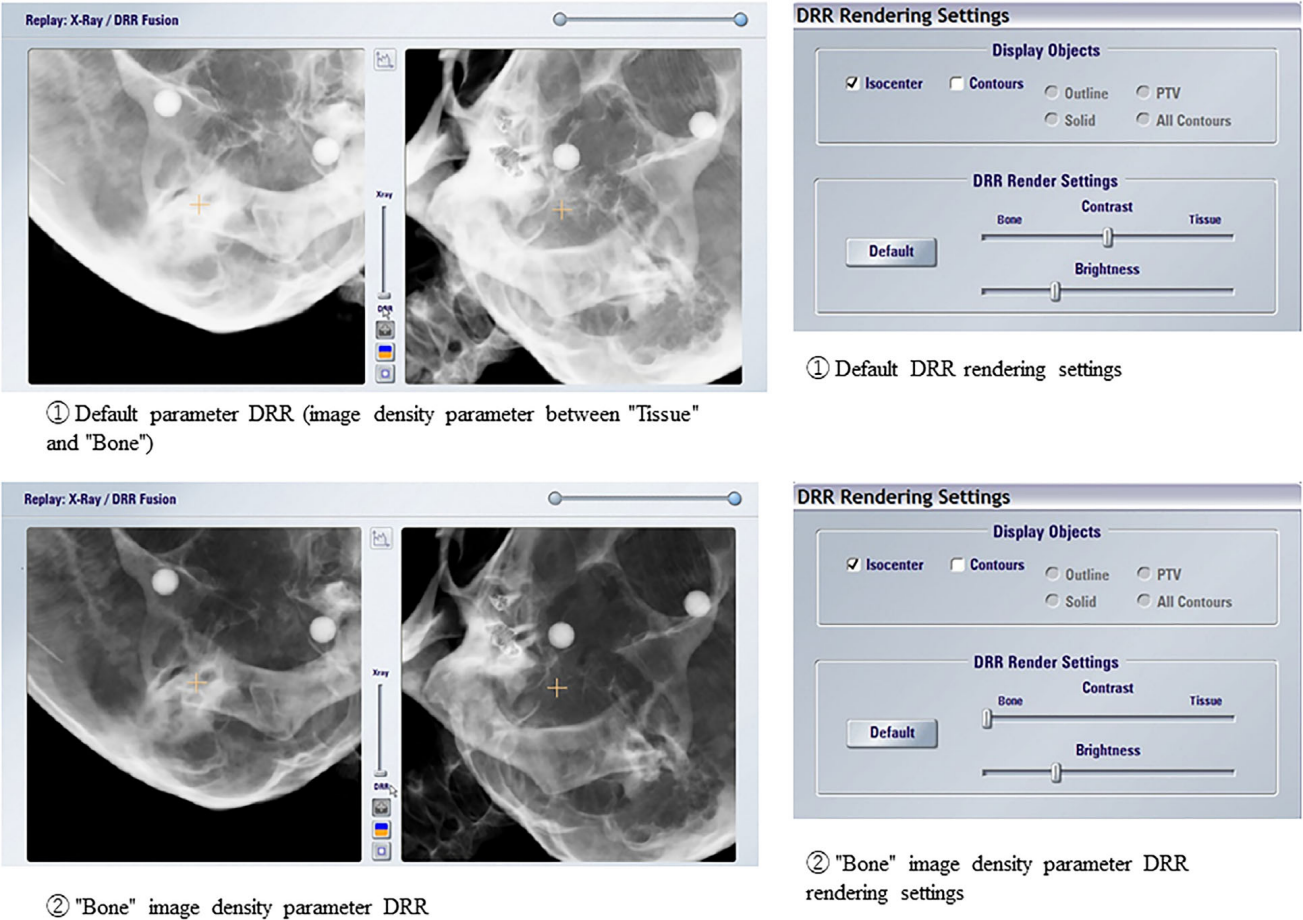
An example of two types of image density adjustment parameters. ① Default parameter digitally reconstructed radiograph (DRR) (image density parameter between “tissue” and “bone”). ② “Bone” image density parameter DRR

**FIGURE 3:**
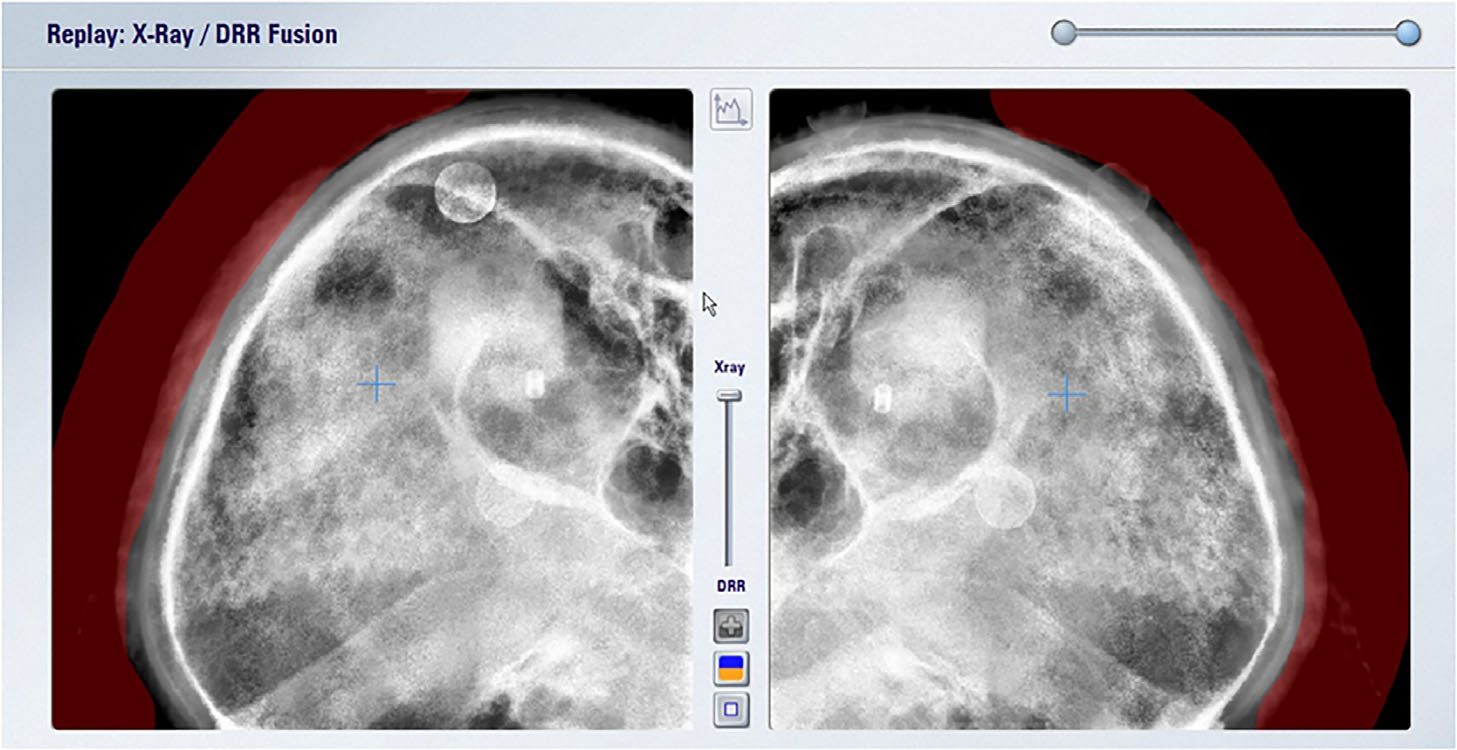
Masking process when the parietal area is included in the ExacTrac X‐ray system. The masking area is red Abbreviation: DRR, digitally reconstructed radiograph.

**FIGURE 4:**
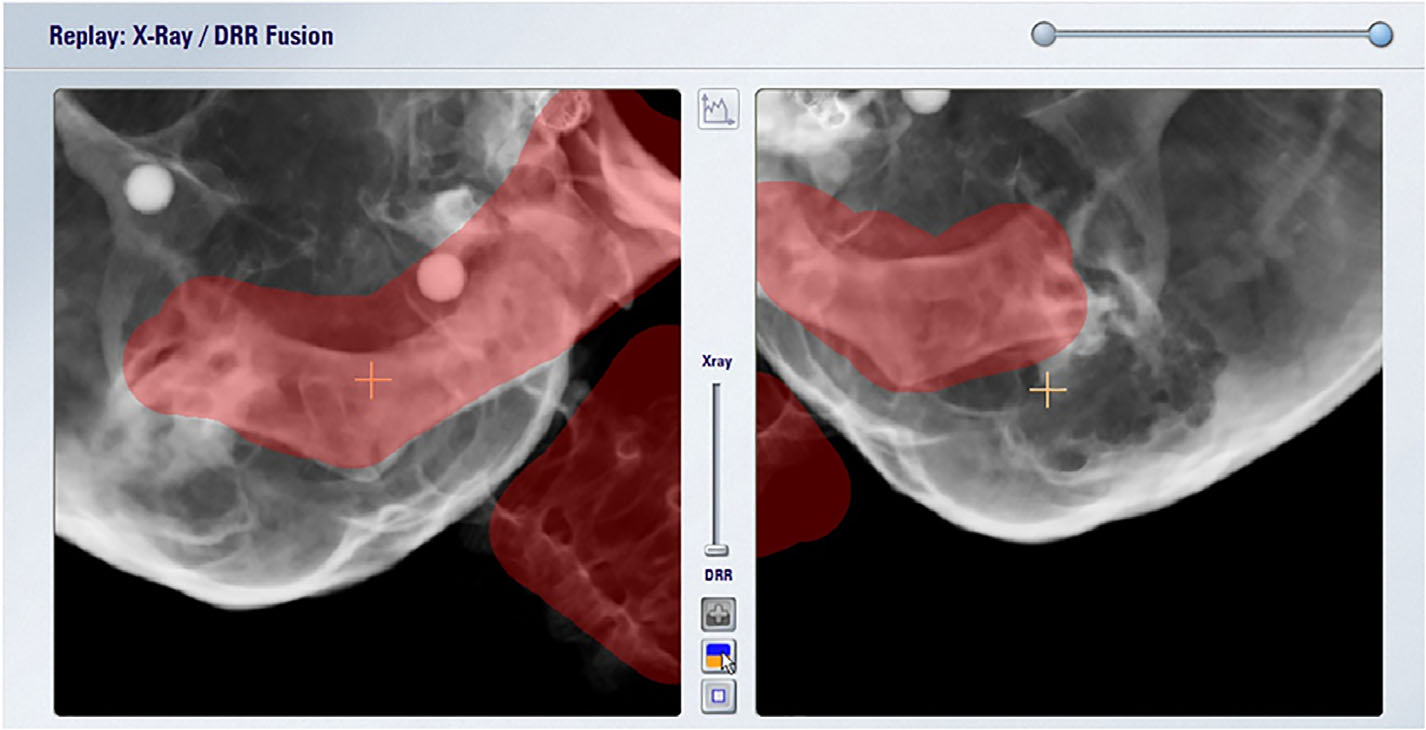
Masking of the mandible and cervical vertebrae within the scanning area of the ExacTrac X‐ray system. The masking area is red Abbreviation: DRR, digitally reconstructed radiograph.

In this study, we evaluated the difference in couch correction values by 6DOF using two types of image density parameters: (1) the default parameter (an image density parameter between “tissue” and “bone”), which is an image density parameter for DRR generation used at many facilities and (2) the bone parameter, which is the steepest contrast parameter used at our hospital. An example of the two image density adjustment parameters is shown in Figure 2. When creating DRRs, many facilities mask the scalp portion during position matching in cases where the tumor is located at the top of the head and the scalp is thick. Therefore, in the case of DRR creation for (1), masking was performed when the parietal area was included within the imaging range of the ETX (Figure 3). In contrast, in the DRR creation in (2), masking was not performed because the scalp was not depicted in the DRR due to the steep contrast. However, when the mandible and cervical vertebrae were included in the range of the ETX, masking was performed for both (1) and (2) DRRs (Figure 4).

### 2.6 Evaluation method

The standard of couch correction resulted from image matching of X‐ray images taken with the DRR and ETX in (2). In the translational direction, we evaluated the difference in image matching results between (1) and (2) according to the three axes (L‐R, S‐I, and A‐P directions) and the three‐dimensional (3D) directions, respectively. The calculation method for the 3D direction was calculated using the following formula:

$$\begin{array}{ccc} & & \mathrm{Three}-\mathrm{dimensional}\;\mathrm{directions}\\ & & =\sqrt{\left(\mathrm{the}\;\mathrm{difference}\;\mathrm{in}\;\mathrm{the}\;\mathrm{correction}\;\mathrm{value}\;\mathrm{of}\;{\mathrm{vertical}}^{2}\right)+\left(\mathrm{the}\;\mathrm{difference}\;\mathrm{in}\;\mathrm{the}\;\mathrm{correction}\;\mathrm{value}\;\mathrm{of}\;{\mathrm{longitudinal}}^{2}\right)+\left(\mathrm{the}\;\mathrm{difference}\;\mathrm{in}\;\mathrm{the}\;\mathrm{correction}\;\mathrm{value}\;\mathrm{of}\;{\mathrm{lateral}}^{2}\right)} \end{array}$$

For the rotational direction, we evaluated the difference in position matching results between (2) and (1) according to the three rotational axes of yaw, roll, and pitch directions, respectively. The evaluation images used in this study were the first X‐ray image at the 0° couch and the first X‐ray image at the actual irradiation couch angle among the X‐ray images taken by the ETX. Therefore, the evaluation images used in this study were not X‐ray images immediately before the final irradiation. The amount of displacement between the X‐ray images of the DRR and ETX was not within 0.5 mm in the translational direction and 0.5° in the rotational axis direction. However, after correcting the couch with 6DOF based on the X‐ray images captured by the DRR and ETX in (2), the images were matched again using the X‐ray images captured by the DRR and ETX in (2). The evaluation images met our acceptable values (within 0.5 mm in the translational direction and 0.5° in the rotational direction). The evaluation images used in this study exclude the results of images taken with the DRR and ETX in (2) at the time of matching, which were within 0.5 mm in the translational direction and 0.5° in the rotational axis direction.

**FIGURE 5:**
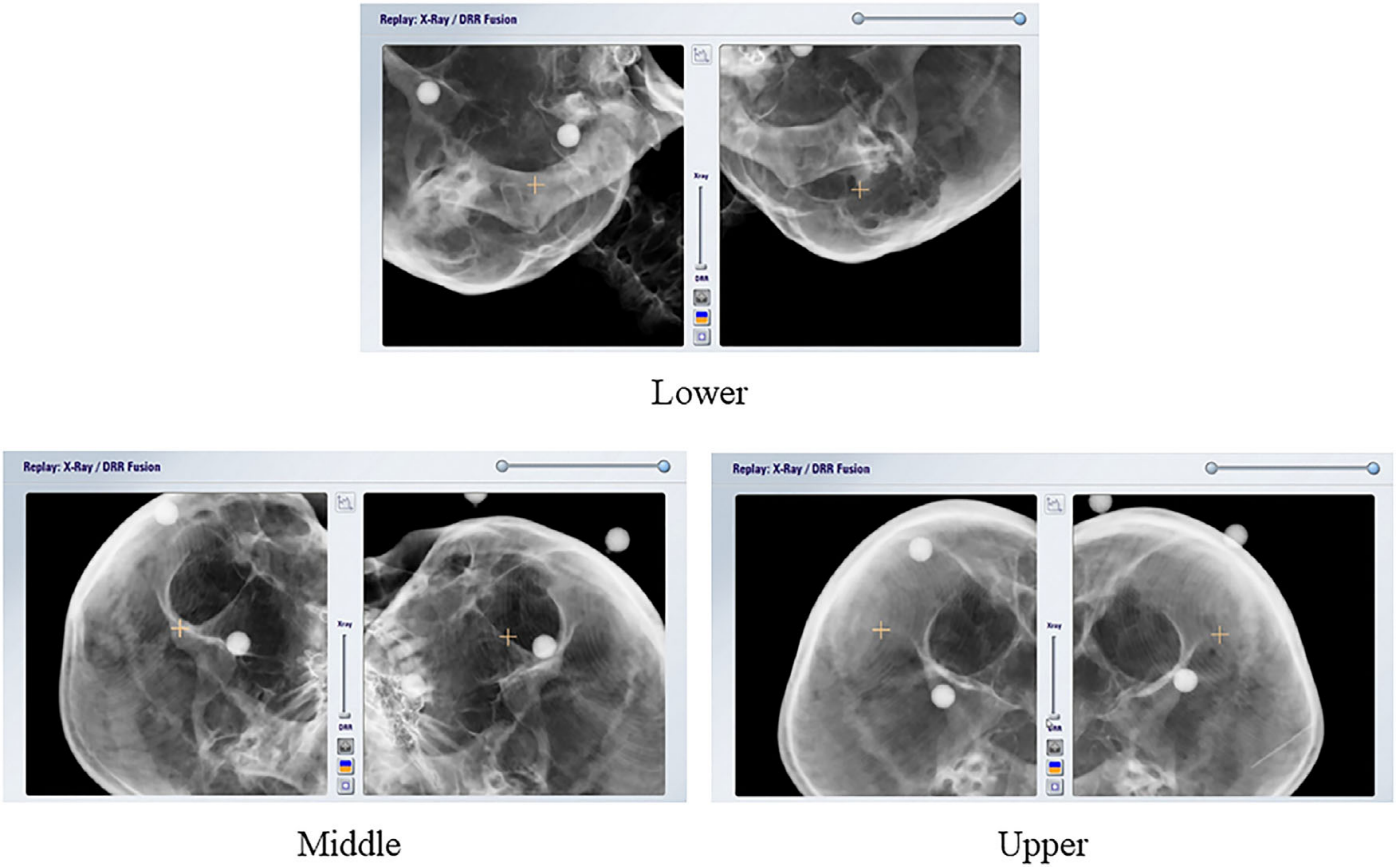
Three classifications of brain metastases according to the region of occurrence (lower, middle, and upper) Abbreviation: DRR, digitally reconstructed radiograph.

In this study, we classified brain metastases into three categories (lower, middle, and upper) according to the area of occurrence. Figure 5 shows an example of the three classifications based on the location of brain metastasis. The number of cases per region of occurrence was 10 (lower), 12 (middle), and 10 (upper). In addition, we evaluated the difference in the results of image matching by the DRR between (1) and (2) of the couch correction value by 6DOF according to the couch angle. The Mann–Whitney *U*‐test was used to evaluate significant differences in translational and rotational directions according to the site of occurrence. A statistically significant difference was determined by a *p*‐value of <0.05.

## 3 RESULTS

**FIGURE 6:**
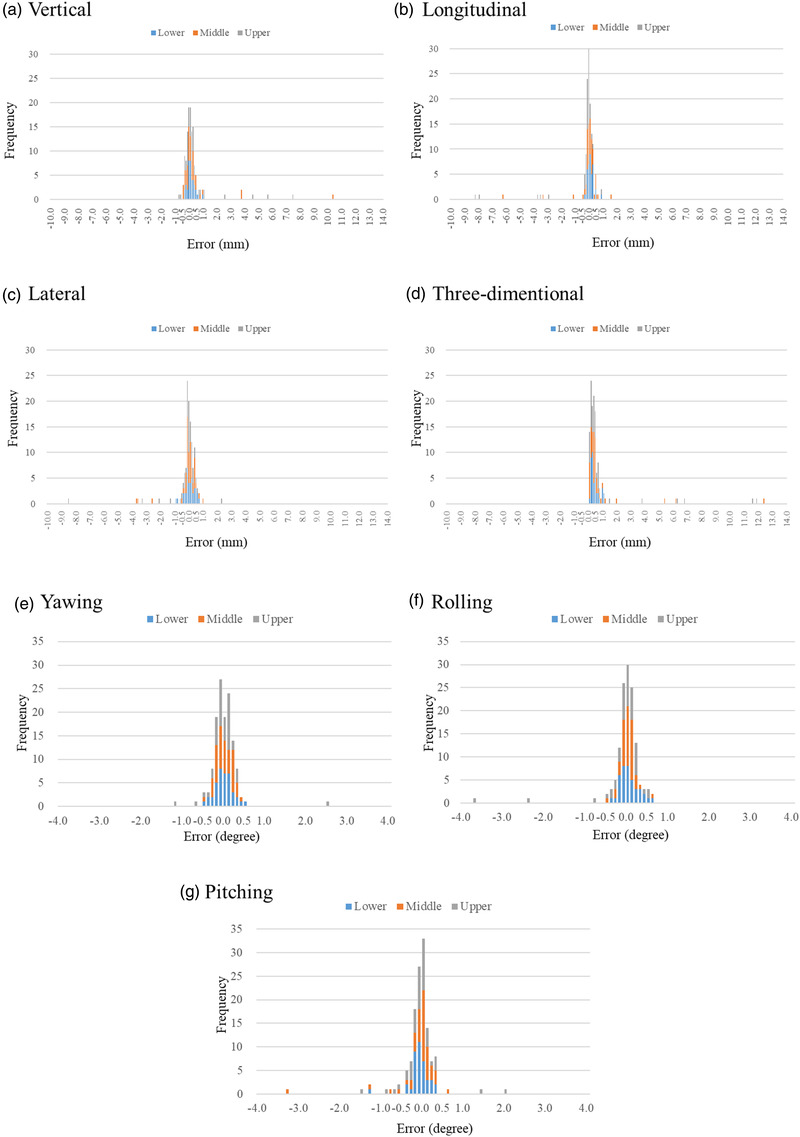
Differences in the couch correction values by six degrees of freedom (6DOF) using two different image density parameters according to the three categories of brain metastasis regions (lower, middle, and upper)

Figure 6 shows the differences in the couch correction values by 6DOF using two different image density parameters according to the three categories of brain metastasis regions (lower, middle, and upper). The percentage of patients with at least one couch angle of 0.5 mm in the translational direction or 0.5° or more in the rotational direction and 1.0 mm or more in the translational direction or 1.0° or more in the rotational direction, according to the three categorized regions of brain metastasis occurrence, is also shown. Among the 10 patients with brain metastases in the lower region, seven (70%) had a translational direction of 0.5 mm or more or a rotational direction of 0.5° or more, and three (30%) had a translational direction of 1.0 mm or more or a rotational direction of 1.0° or more. Of the 12 patients with brain metastases in the middle region, eight (66.6%) had a translational direction of 0.5 mm or more or a rotational direction of 0.5° or more, and four (33.3%) had a translational direction of 1.0 mm or more or a rotational direction of 1.0° or more. Of the 10 patients with brain metastases in the upper region, nine (90%) had a translational direction of 0.5 mm or more or a rotational direction of 0.5° or more, and three (30%) had a translational direction of 1.0 mm or more or a rotational direction of 1.0° or more. Of the 32 patients, 24 (75%) had a translational direction of 0.5 mm or more or a rotational direction of 0.5° or more, and 10 (31%) had a translational direction of 1.0 mm or more or a rotational direction of 1.0° or more. The results of the Mann–Whitney *U*‐test are summarized in Table 1. Significant differences were found only in the longitudinal direction between the upper and middle regions and between the upper and lower regions.

**TABLE 1:** The results of the Mann–Whitney *U*‐test

	Translation shift			
	Vertical	Longitudinal	Lateral	Three dimensional
Lower vs. middle	0.674	0.340	0.381	0.986
Lower vs. upper	0.882	<0.001	0.217	0.975
Middle vs. upper	0.734	0.001	0.696	0.879

	Rotational shift		
	Yaw direction	Roll direction	Pitch direction
Lower vs. middle	0.979	0.815	0.208
Lower vs. upper	0.612	0.422	0.872
Middle vs. upper	0.561	0.676	0.297

*Note*: Significant differences were found only in the longitudinal direction between the upper and middle regions and between the upper and lower regions.

**FIGURE 7:**
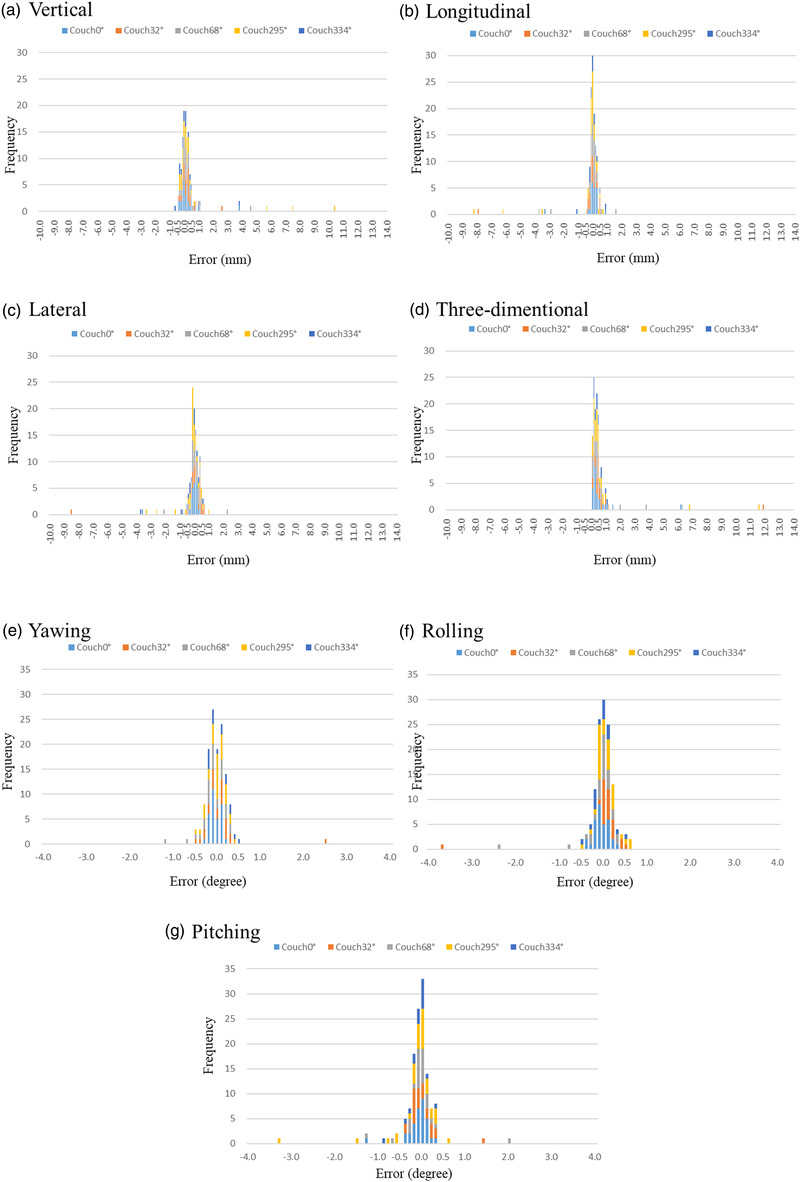
Differences in the couch correction values by six degrees of freedom (6DOF) using two different image density parameters according to the five table angles (couch 0°, 32°, 68°, 295°, and 334°). Couch32°: the average (minimum to maximum) couch angles were 32° (15°–45°). Couch68°: the average (minimum to maximum) couch angles were 68° (55°–80°). Couch295°: the average (minimum to maximum) couch angles were 295° (285°–315°). Couch334°: the average (minimum to maximum) couch angles were 334° (330°–345°)

Figure 7 shows the differences in the couch correction values by 6DOF using two different image density parameters according to the five table angles (couch 0°, 32°, 68°, 295°, and 334°). The percentages of cases with a translational direction of 0.5 mm or more or a rotational direction of 0.5° or more and a translational direction of 1.0 mm or more or a rotational direction of 1.0° or more for each couch angle were described. Of the 24 cases with a mean couch angle of 32° (minimum couch angle of 15° to maximum couch angle of 45°), 11 (46%) had a translational or rotational direction of 0.5 mm or more, and two (8%) had a translational or rotational direction of 1.0 mm or more or 1.0° or more. Of the 27 cases with a mean couch angle of 68° (minimum couch angle of 55° to maximum couch angle of 80°), eight (30%) had a translational direction of 0.5 mm or more or a rotational direction of 0.5° or more, and three (11%) had a translational direction of 1.0 mm or more or a rotational direction of 1.0° or more. Of the 32 cases with a mean couch angle of 295° (minimum couch angle of 285° to maximum couch angle of 315°), 13 (41%) had a translational or rotational couch angle of 0.5 mm or more, and four (13%) had a translational or rotational couch angle of 1.0 mm or more or 1.0° or more. Of the 16 cases with a mean couch angle of 334° (minimum couch angle of 330° to maximum couch angle of 345°), six (38%) had a translational direction of 0.5 mm or more or a rotational direction of 0.5° or more, and three (19%) had a translational direction of 1.0 mm or more or a rotational direction of 1.0° or more. Of the 131 cases at all couch angles, 46 (35%) had a translational direction of 0.5 mm or more or a rotational direction of 0.5° or more, and 15 (11%) had a translational direction of 1.0 mm or more or a rotational direction of 1.0° or more (Figures 6 and 7).

**FIGURE 8:**
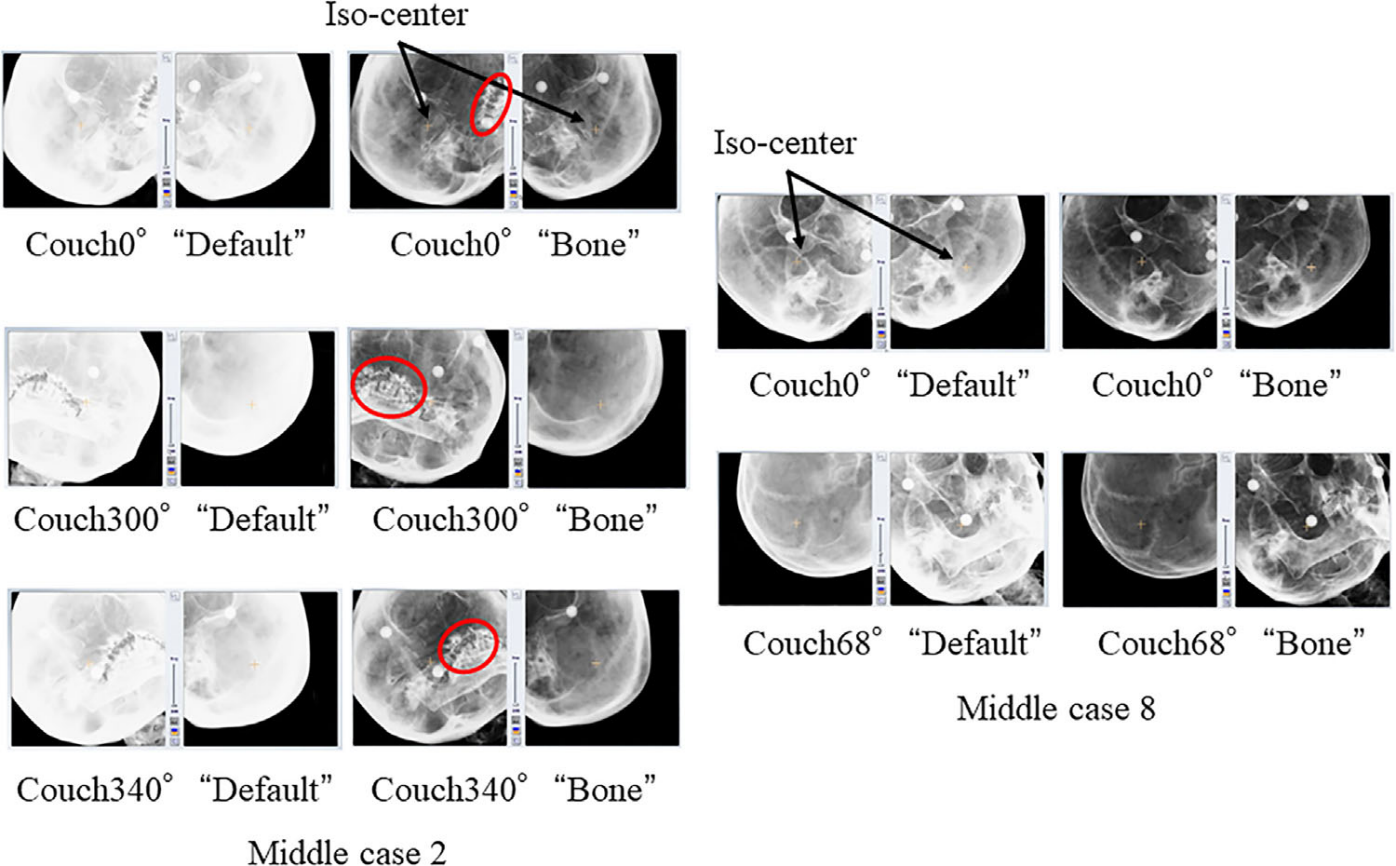
Two cases of middle region of brain metastasis with a translational direction of 1.0 mm or more or rotational direction of 1.0° or more. The digitally reconstructed radiograph (DRR) of the couch angle that is misaligned with the result of position matching is shown with two DRR parameters, “default” and “bone.” Red circles indicate the areas of metal artifacts caused by dentures

**FIGURE 9:**
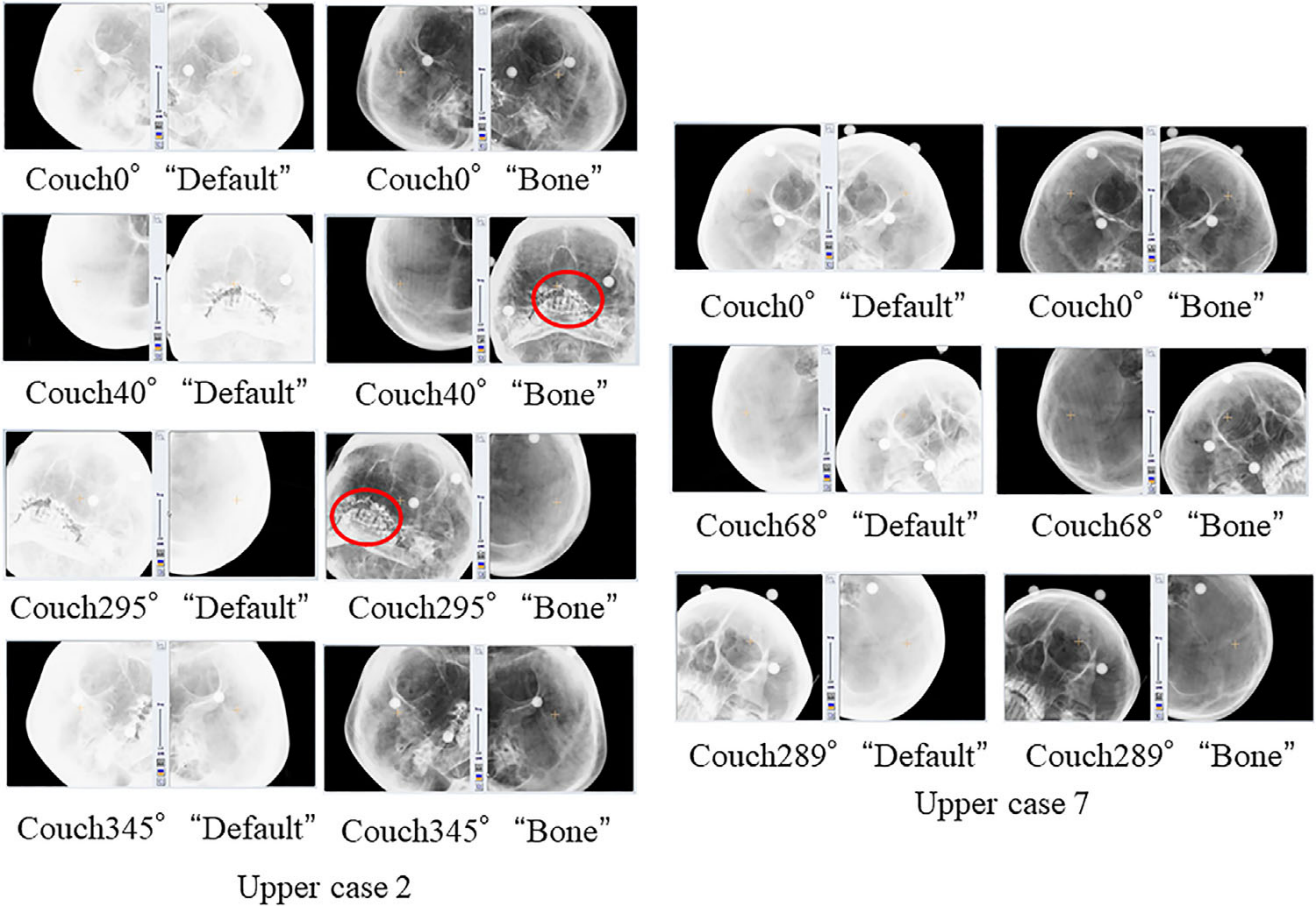
Two cases of upper region of brain metastasis with a translational direction of 1.0 mm or more or rotational direction of 1.0° or more. The digitally reconstructed radiograph (DRR) of the couch angle that is misaligned with the result of position matching is shown with two DRR parameters, “default” and “bone.” Red circles indicate the areas of metal artifacts caused by dentures

Figure 8 shows the DRRs of two image density parameters in the translational direction of 1.0 mm or more or the rotational direction of 1.0° or more for two cases of middle brain metastases. Figure 9 shows the DRRs of two image density parameters in two cases of brain metastasis in the upper region for the translational direction of 1.0 mm or more and a rotational direction of 1.0° or more.

## 4 DISCUSSION

This study investigated the effects of two different image density adjustment parameters on the image matching results at 6DOF using the ETX. No characteristic effects associated with the region of brain metastasis were observed. In addition, the difference in the correction value of the couch by 6DOF corresponding to the couch angle tended to be larger in other couches than in the 0° couch.

Koubuchi et al.^11^ reported that the accuracy of the ETX retained a high positional accuracy of 0.5 mm even after rotation of the couch regardless of positional correction and concluded that the positional accuracy was sufficient for brain localization.^11^ However, they used an ahead phantom,^11^ and because the phantom does not include the torso, the load on the couch may be different from the actual load on the patient. In contrast, Tanaka et al.^16^ evaluated and reported the uncertainty of patient positioning in noncoplanar SRS or stereotactic radiation therapy of intracranial lesions with 6DOF using the ETX. According to Tanaka et al., the uncertainty of patient positioning was 1.0–2.0 mm after couch rotation. However, their report did not determine whether the patient positioning error results from intra‐fractional motion or a registration error caused by the position matching software included with the ETX.

In this study, we hypothesized that patient positioning errors due to differences in image density parameters would lead to registration errors in the ETX registration software. Two types of DRRs were created: one with the default parameters used in many facilities and the other with the “bone” image density parameters, which are the steepest contrast parameters used in our hospital. We believe that evaluating the differences in the couch corrections due to 6DOF between the two DRRs will update the clinical knowledge base and provide helpful information for ETX users. ETX users who create DRRs with default parameters may misrecognize the scalp area as a bone if the tumor is located at the top of the head. Therefore, many facilities exclude the scalp from position matching by masking it. However, this masking process must be performed manually by a radiologist in charge of radiation therapy, which leads to an increase in treatment duration.

In contrast, in DRRs created using the “bone” image density parameter the scalp area is not depicted on the DRR because of the steep contrast. Therefore, position matching can be performed without masking, which can lead to a reduction in the treatment time of approximately 5 min. However, an extended treatment time may impair inpatient fixation accuracy.^19^

As shown in Figure 2, the contrast setting was set to a level between “tissue” and “bone” in the default parameter. The contrast setting did not reflect the differences between the cases. Therefore, the difference in contrast between the DRRs of the default parameters shown in Figure 6, middle case 2, and Figure 7 and the DRRs of the default parameters shown in Figure 2 would have occurred in different cases. The ETX technical user guide^18^ clearly states that it is unnecessary to change the default values in most cases. However, there is a warning about changing the contrast and brightness of the position matching image results. In this study of brain metastases, the difference in DRR between parameters (1) and (2) was significant even though the skull is a site with a relatively steeper bone and tissue contrast when compared with that from other sites.

In a previous study, the difference in position matching results between the head, neck, and pelvic regions using the ETX has been reported.^20^ Wu et al.^20^ concluded that the pelvic region had better bone‐to‐tissue contrast and edge detection effectiveness than the head and neck regions, resulting in better image matching. From the DRRs shown in Table 1 of this study, we believe that the effectiveness of bone‐to‐tissue contrast and edge detection in DRRs in (2) is the reason for the difference in results between DRRs in (1) and (2) and the ETX image matching. In addition, in the ETX image matching, the amount of misalignment resulting from moving the DRR in the translational or rotational direction is calculated for the X‐ray image captured by the ETX.^18^ When calculating the amount of misalignment, multiple DRRs are generated from the treatment plan CT image captured by the ETX in advance by entirely using the calculation function of the graphics board and performing high‐speed rendering processing image matching. Therefore, we believe that DRRs with a steep contrast difference, such as the DRR in (2), showed good image matching results. If present, it is also common for denture metal artifacts to be masked as they do not reflect the appropriate CT number. However, in Figure 6 (middle case 2) and Figure 7 (upper case 2), there are metal artifacts near the isocenter if masking is performed. In this case, it will be difficult to perform proper position matching due to the lack of bone structure. However, we believe that the presence of denture metal artifacts caused a disadvantage in patient position matching by increasing the overall density value in the DRR of the default parameter, resulting in an image with less contrast difference. In recent years, an algorithm for metal artifact reduction (MAR) has been developed, and there are several reports of its clinical application.^21^, ^22^ Currently, however, MAR is still not available in all facilities where radiotherapy is performed.

There are two methods of ETX calibration, X‐ray calibration and position calibration, and the overall accuracy of the ETX is confirmed by accurately performing both the methods. However, both calibration methods are performed at a couch angle of 0° and not at any other angle. Therefore, it is possible to include uncertainty in the generation of multiple DRR patterns from the treatment plan CT imported into the ETX by the fast rendering process when the couch angle is not 0° compared to the couch angle of 0°.

In our hospital, we use the phantom attached to the ETX to check the center position of the laser and the ETX in the treatment room as a part of daily quality control (QC). The tolerance is 0.3 mm or less in the direction of coaxial movement of the three axes, and the action level is set if the tolerance exceeds 0.5 mm. The response to the action level is to adjust the ETX calibration of the X‐rays and the position. In addition, as part of the monthly QC, the accuracy of the center position of the radiation isocenter and the ETX is checked, and the difference between the two is confirmed to be within 1 mm. If the difference is greater than 1 mm, we set an action level. The response to the action level is to adjust the laser in the treatment room and recalibrate the ETX calibration of the X‐rays and position. Therefore, we believe that we have confirmed the same accuracy control of the ETX, as reported in previous studies.

In addition, the ETX technical user guide^18^ describes five ways to improve the auto‐fusion results of poor images: setting the initial value of auto‐fusion by performing manual fusion, setting the volume of interest (VOI) on the relevant anatomical structure, changing the maximum area of fusion, setting the region of interest of the X‐ray image, and changing the look‐up table value of the DRR calculation. First, in manual fusion, the DRR and the superimposed X‐ray image are visually checked, and the DRR is moved to the position that best matches the X‐ray image, which enables position matching in three axes. In the ETX, the vertebrae and ribs are easily confused and are not suitable for sites where submillimeter position matching is required, such as brain localization. Next, setting the VOI on the anatomical structure is a method to exclude a part of the bone structure in the CT image in the image position matching. It is used for mobile bone structures that move independently from the target area such as the clavicle and mandible or when the body's position in the planning CT image differs from the curvature of the spine in the pre‐treatment position matching. However, since it is only possible to define the VOI setting as a cube in the treatment planning CT image, it is unlikely to directly impact the results of this study. In the improvement method of changing the maximum area of fusion, it is also described that the maximum area of the image fusion target should be reduced if the result of the auto‐fusion is wrong and needs to be corrected significantly. This process is particularly effective when the patient setup is nearly perfect before image fusion is performed. In this study, 5 mm was used as the initial setting for the maximum range. However, the maximum range of positioning at the actual irradiation couch angle after positioning at a couch angle of 0° is considered to be approximately 3 mm, which is a reasonable value considering the uncertainty of the couch accuracy reported by previous studies.^11^, ^16^

Regarding the setting of the region of interest in the X‐ray image, the auto‐fusion exclusion region is set in this study as shown in Figures 3 and 4, but we believe that improving the setting method is still possible. However, we also believe that the setting of the exclusion region does not dramatically change the results of this study. The change in the look‐up table value in the DRR calculation is the concentration parameter of the DRR and is the most influential part of the results of this study. Oh et al.^23^ conducted a study of 107 cases of SRS and performed an off‐line review using both ETX with 6DOF and six‐dimensional (6D)‐CBCT with the patients uniformly fixed. In their report, they concluded that the discrepancy in residual setup error was minor but should still be considered. Since the present study was conducted using only ETX with 6DOF, we believe that it should be compared with CBCT in the future. ETX with 6DOF can estimate the 3D position matching result from the 2D position matching result, and it can be compared with the true 3D image result by comparing with CBCT.

In recent years, the optical surface imaging (OSI) system has been attracting attention as a position matching device without radiation exposure, and its clinical application in brain localization has begun.^24^ A study reporting that a dedicated real‐time monitoring system for detecting intra‐fractional head motion in intracranial radiotherapy using a pressure sensor has been developed as a position matching device without exposure has been conducted. However, it is not a commercially available hardware.^25^ In the future, we believe that it will be important to combine the OSI system and pressure sensor with the ETX system to monitor motion during treatment and further improve treatment outcomes by reducing margins.

In conclusion, this study shows the usefulness appropriate DRR parameters for each case, instead of using the default settings. Using appropriate DRR parameters, accurate position matching results can be obtained. Furthermore, accurate matching results can reduce the number of IGRT shots, leading to a reduction in the imaging dose.

## CONFLICT OF INTEREST

The authors declare no conflict of interest.

## ETHICS STATEMENT

The study design was approved by the Clinical Research Ethics Review Committee of Tokushima University Hospital (approval number: 3434).

## AUTHOR CONTRIBUTIONS

Kanako Sakuragawa, Motoharu Sasaki, Takeshi Kamomae, and Hitoshi Ikushima carried out the conceptual design of the study. Kanako Sakuragawa, Ryosuke Kasai, and Akimi Kajino collected the data. Michihiro Yokoishi performed the data analysis. All authors discussed the interpretation of the submitted papers, wrote or critically revised their articles on important intellectual content, and have given final approval to the submitted papers.
